# Urinary Proteomic Shifts over Time and Their Associations with eGFR Decline in Chronic Kidney Disease

**DOI:** 10.3390/biom15010045

**Published:** 2025-01-01

**Authors:** Zhalaliddin Makhammajanov, Kamila Nurlybayeva, Zikrillo Artikov, Pavel Tarlykov, Mohamad Aljofan, Rostislav Bukasov, Duman Turebekov, Syed Hani Abidi, Mehmet Kanbay, Abduzhappar Gaipov

**Affiliations:** 1 Department of Biomedical Sciences, School of Medicine, Nazarbayev University, Astana 010000, Kazakhstan; 2 School of Medicine, Koc University, Istanbul 34450, Turkey; 3 Department of Therapy, National Scientific Medical Center, Astana 010000, Kazakhstan; 4 Department of Proteomics and Mass Spectroscopy, National Center for Biotechnology, Astana 010000, Kazakhstan; 5 Department of Chemistry, School of Sciences and Humanities, Nazarbayev University, Astana 010000, Kazakhstan; 6 Department of Internal Medicine, Astana Medical University, Astana 010000, Kazakhstan; 7 Division of Nephrology, Department of Internal Medicine, Koc University, Istanbul 34450, Turkey; 8 Department of Medicine, School of Medicine, Nazarbayev University, Astana 010000, Kazakhstan; 9 Clinical Academic Department of Internal Medicine, University Medical Center, Astana 010000, Kazakhstan

**Keywords:** biomarkers, VTDB, CD44, FBN1, urinary proteomics, proteinuria, chronic kidney disease

## Abstract

Chronic kidney disease (CKD) is a progressive condition characterized by declining renal function, with limited biomarkers to predict its progression. The early identification of prognostic biomarkers is crucial for improving patient care and therapeutic strategies. This follow-up study investigated urinary proteomics and clinical outcomes in 18 CKD patients (stages 1–3) and 15 healthy controls using liquid chromatography–mass spectrometry and Mascot-SwissProt for protein identification. The exponentially modified protein abundance index (emPAI) was used for peptide quantification. Regression analyses were used to evaluate relationships between urinary proteins and the estimated glomerular filtration rate (eGFR), adjusting for proteinuria. At baseline, 171 proteins (median emPAI 86) were identified in CKD patients, and 271 were identified (median emPAI 47) in controls. At follow-up, 285 proteins (median emPAI 44.8) were identified in CKD patients, and 252 were identified (median emPAI 34.2) in controls. FBN1 was positively associated with eGFR, while FETUA showed a significant negative correlation at baseline. At follow-up, VTDB shifted from a negative baseline to a positive association with eGFR over time. CD44 and FBN1 shifted from a positive baseline to a negative association over time. These findings highlight VTDB, FBN1, and CD44 as potential prognostic biomarkers, providing insights into CKD progression and therapeutic targets.

## 1. Introduction

Chronic kidney disease (CKD) is a rapidly growing non-communicable disease of global concern. CKD is responsible for over 1.2 million annual deaths, and projections indicate that by 2040, it could become the fifth most common cause of death worldwide [1,2]. CKD is a primary cause of morbidity and mortality, particularly among older adults [2]. Over 80% of adults with CKD also have high blood pressure, which significantly increases the risk of cardiovascular disease and accelerates CKD progression [3]. The limited success of current treatments is mainly due to late detection, underscoring the importance of early diagnosis and a deeper understanding of CKD progression to alleviate its health and economic burden [4].

Proteinuria remains the primary biomarker and risk indicator for chronic and diabetic kidney diseases. The presence of proteins in urine, known as proteinuria, may arise from various underlying mechanisms, including pre-renal, renal, and post-renal conditions [5]. Renal proteinuria is caused by compromised glomerular filtration and/or the impaired reabsorption of proteins by proximal tubular epithelial cells (PTECs). The excessive reabsorption of filtered proteins can be toxic to tubular cells, leading to inflammation, interstitial fibrosis, tubular atrophy, and, ultimately, progression to end-stage kidney disease (ESKD) [6]. Consequently, patients with high proteinuria levels often experience a faster decline in kidney function. However, not all patients follow this pattern; some exhibit slower disease progression despite high proteinuria levels, suggesting variability in outcomes that are poorly understood [7]. The physical and chemical properties of urinary proteins, such as their cell origin, biological function, molecular weight, and molecular moieties bound to proteins or triggers for secretion, could explain these differences [6,8]. Identifying high-risk proteins associated with kidney function and proteinuria could be valuable prognostic markers for CKD progression.

Despite using proteinuria as a clinical marker, its limited specificity and sensitivity in predicting disease progression highlight the need for additional and more specific urinary protein biomarkers [9]. In our previous cross-sectional study on urinary biomarkers, several proteins, including FETUA, VTDB, B2MG, AMBP, LV39, CERU, and A1BG, were significantly associated with the estimated glomerular filtration rate (eGFR) in CKD patients [10]. However, their long-term associations with eGFR remain unknown. Urinary proteomics has the potential to enhance our understanding of CKD progression by offering insights into protein changes that may serve as biomarkers for disease progression. While previous proteomic studies have identified various potential biomarkers for CKD, many have focused on spot urine [11] or blood samples [12], which may not fully represent the complexity and variability of the disease. Although some longitudinal urinary proteomics studies have examined follow-up eGFR data [13,14], they often have not focused on the follow-up of proteomic profiles. In this follow-up study, we provide a comprehensive longitudinal analysis comparing baseline and follow-up urinary proteomes in CKD patients and healthy controls, assessing associations with changes in eGFR over time. Through this analysis, we aim to identify prognostic biomarkers that offer more reliable predictions of CKD progression, and which contribute to developing improved diagnostic strategies.

## 2. Materials and Methods

### 2.1. Study Population and Design

This follow-up study involved a cohort of 18 patients with early-stage CKD and a healthy control group. The recruitment of study participants took place at the National Scientific Medical Center (NSMC, Astana, Kazakhstan) between March 2020 and December 2022. The study is registered as a part of a clinical trial on ClinicalTrials.gov (ID: NCT04311684) [15]. Prior to any procedures, all participants were thoroughly informed about the study and provided written consent.

Participants were confirmed to be free of COVID-19 upon enrollment. Eligibility criteria for the patient group included a diagnosis of CKD stages 1–3, following the KDIGO 2012 guidelines [16], with the primary cause attributed to glomerular diseases and proteinuria exceeding 150 mg per 24 h. Individuals were excluded if they were younger than 18 or older than 70 years, pregnant, or had a history of cancer, infections, or other severe comorbid conditions. Glomerular diseases were diagnosed based on a clinical assessment, including eGFR and proteinuria measurements, without confirmation by kidney biopsy.

### 2.2. Laboratory Analysis and Sample Collection

The laboratory staff conducting the analyses were blinded to the participant’s clinical status until the study was completed, ensuring unbiased sample processing. Blood and urine specimens were collected independently of patient outcomes. Routine blood tests included assessments of serum creatinine, urea, uric acid, cholesterol, total protein, glucose, and a complete blood count for all subjects. A comprehensive 24-h urine analysis was performed to measure the total protein rate during participant enrollment. The estimation of 24-h proteinuria levels was performed by multiplying the protein concentration in urine (in g/L) by the total volume of urine collected (in mL) over 24 h. The formula is expressed as follows: *A = n × v*

Here,

*A* = 24-h protein excretion (mg/24 h);

*n* = protein concentration in urine (g/L);

*v* = urine volume (mL).

Routine biochemical tests were carried out on a Cobas Integra 400 plus analyzer (Roche Diagnostics, Indianapolis, IN, USA). The eGFR was calculated using the 2021 CKD-EPI formula [17]. Demographic and clinical data, including medical history and comorbid conditions, were obtained through direct interviews and verified against medical records.

### 2.3. Proteomic Analysis by Mass Spectrometry

For subsequent proteomics analysis, extra 24-h urine samples were collected, frozen at −80 °C, and stored until analysis. Protein isolation was performed using an acetone precipitation method, modified according to the procedure by [18]. Isolated proteins were dissolved in a 25 mM ammonium bicarbonate (NH_4_HCO_3_) buffer and kept at −80 °C until further use. Per the manufacturer’s protocol, protein concentrations were determined with a NanoDrop 1000 spectrophotometer (Thermo Fisher Scientific, Waltham, MA, USA).

The protein samples were reduced, alkylated, and enzymatically digested using trypsin (20 ng/μL) at 37 °C overnight. Following digestion, peptides were purified and concentrated using ZipTip C18 pipette tips (Millipore, Burlington, MA, USA). The desalted peptides were dried using a centrifugal evaporator (Eppendorf, Hamburg, Germany) and reconstituted in 10 μL of 0.1% trifluoroacetic acid.

Peptide separation was achieved through nanoflow reversed-phase liquid chromatography–tandem mass spectrometry (LC-MS/MS). Chromatographic separation was achieved by a Dionex HPLC system with an Acclaim PepMap100 C18 pre-column for trapping, followed by an Acclaim PepMap100 C18 analytical column (Thermo Fisher Scientific, Waltham, MA, USA) for peptide separation using a 75-minute acetonitrile gradient at a flow rate of 0.3 μL/min. Ionization was performed using a captive spray source (capillary voltage 1500 V, dry gas 3.0 L/min, dry temperature 150 °C) coupled to an Impact II ESI-QUAD-TOF mass spectrometer (Bruker Daltonics, Bremen, Germany). Full MS scans were collected at a rate of 2.0 Hz, with subsequent MS/MS scans acquired for peptide identification.

MS/MS data were processed using Data Analysis software (version 3.4, Bruker Daltonics, Bremen, Germany) and exported in Mascot Generic Format. The files were then searched using Mascot (version 2.6.1, Matrix Science, London, UK) against the Swiss-Prot database (release February 2021), restricted to human proteins (20,396 sequences). Search parameters included methionine oxidation as a variable modification and cysteine carbamidomethylation as a fixed modification, allowing for up to two missed cleavages. Mass tolerances were set at 100 ppm for MS and 0.05 Da for MS/MS. The false discovery rate (FDR) was controlled at 1%, and a decoy database search was used for FDR estimation.

Quantification was performed using the exponentially modified protein abundance index (emPAI) and a label-free quantification approach based on spectral counts of tryptic peptides calculated with Mascot 2.6.1 software [19]. Identified proteins were considered significant at a *p*-value threshold of <0.05. The list of detected proteins is illustrated in Figure 2, with “occurrence (%)” indicating protein detection frequency across samples and distribution patterns among groups, providing an overview of the protein’s presence in the analyzed samples rather than quantitative abundance.

The proteomic dataset was processed using log2 transformation followed by normalization based on mean and slope adjustments to maintain data consistency and reliability for further analysis.

### 2.4. Statistics and Data Visualization

All statistical analyses were performed using Stata MP2 version 18.0 and R version 4.4.1 software. Continuous variables were summarized as the mean ± standard deviation (SD) for normally distributed data or as the median with the interquartile range (IQR) for non-normally distributed data. Categorical variables were reported as frequencies and percentages. The chi-square test was used to compare categorical variables. For group comparisons, the Wilcoxon rank-sum test was applied for non-parametric data, and a two-sided *t*-test was applied for parametric data.

Correlations between the proteomic profiles and eGFR were assessed using Spearman’s rank correlation coefficient. Linear regression models were constructed to evaluate the relationship between protein levels (measured as emPAI) and eGFR. Each protein was analyzed in a separate model, where eGFR was the dependent variable, and the emPAI value of the protein was the primary independent variable. The models were adjusted for proteinuria as a covariate. Additionally, an interaction term between protein levels and the follow-up period was included to determine whether the effect of protein levels on eGFR changed over time.

Results from the regression models were reported as regression coefficients, standard errors, t-statistics, and *p*-values. Power analysis was conducted for the primary outcome measure (eGFR) based on the study sample size of 33 participants (18 CKD patients and 15 controls), yielding a power estimate of 0.99.

All *p*-values reported in the manuscript are unadjusted. Statistical significance was set at a *p*-value threshold of <0.05. To ensure consistency and reproducibility, we verified the accuracy of the reported *p*-values by re-running all statistical analyses during manuscript preparation.

Data visualizations were created using the ggplot2 package 3.5.0 in R 4.4.1 [20]. A heatmap was designed to illustrate the distribution of proteins across the control and CKD groups, comparing baseline and follow-up samples. Line plots were used to show changes in eGFR, KDIGO CKD stages, and 24-h proteinuria from baseline to follow-up. Interaction plots were constructed to explore the association between urinary protein emPAI levels and eGFR at both time points, highlighting statistically significant results and corresponding regression coefficients.

## 3. Results

### 3.1. General Characteristics and Disease Progression

The general characteristics of study participants are presented in Table 1. In the current study, a total of 33 participants (patients = 18 and controls = 15) were included in the final analysis, with a mean age of 37.8 years (SD = 9.9), with 54.5% female (*n* = 18), and a median (IQR) follow-up duration of 368 (250–472) days. Age was matched between groups, with a mean age of 37 years (SD = 8) for the control group and 39 years (SD = 11) for the patient group at baseline. The control group comprised 73% female and 27% male participants, while the patient group comprised 39% female and 61% male participants. In 78% of CKD cases, glomerular disease was the primary cause, leading to laboratory abnormalities such as hypoproteinemia, proteinuria, elevated creatinine, and hyperlipidemia, distinguishing CKD patients from healthy controls. Additionally, CKD patients had a significantly higher prevalence of hypertension (67% vs. 7% in controls) and elevated inflammatory markers (median ESR of 21 mm/h vs. 9.6 mm/h in controls at baseline, *p* < 0.001), highlighting the systemic inflammation associated with CKD.

**Figure 1:**
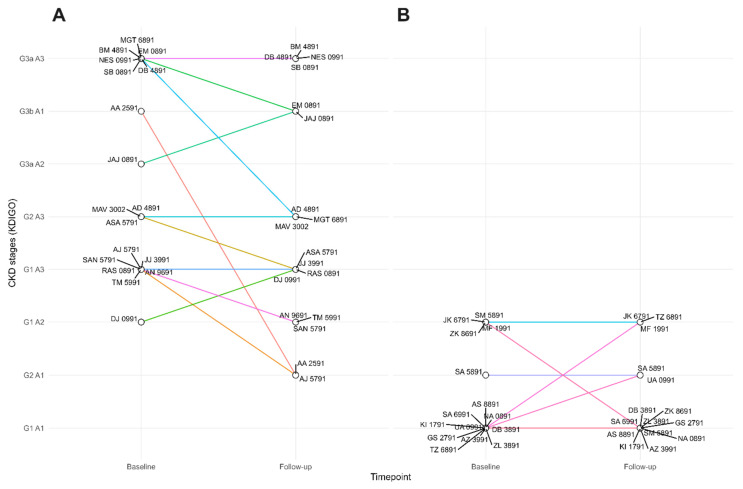
Line plot of CKD stage changes over time in patient (**A**) and control (**B**) groups based on GFR and proteinuria categories, with colour coding according to the KDIGO 2012 guideline [16]. The X-axis displays individual participant IDs to show CKD stage changes from baseline to follow-up, while the Y-axis conveys CKD stages based on increasing risk.

Study participants were characterized using GFR and proteinuria levels according to the KDIGO 2012 guideline (Table 2) [16]. Based on CKD risk stratification using GFR and proteinuria categories, along with colour coding from the KDIGO CKD classification, changes in the CKD stage over time were evaluated and are depicted in Figure 1. Only two patients exhibited CKD progression during the follow-up period, with one moving from G1 A2 to G1 A3 and another from G3a A2 to G3b A1. Most patients remained stable in their CKD risk category or showed improvements during the follow-up period (Table 2 and Figure 1), even though some patients experienced declines in eGFR (Supplementary Figure S1) and/or increases in proteinuria levels (Supplementary Figure S2).

### 3.2. Proteomic Profiles of Study Population

**Figure 2:**
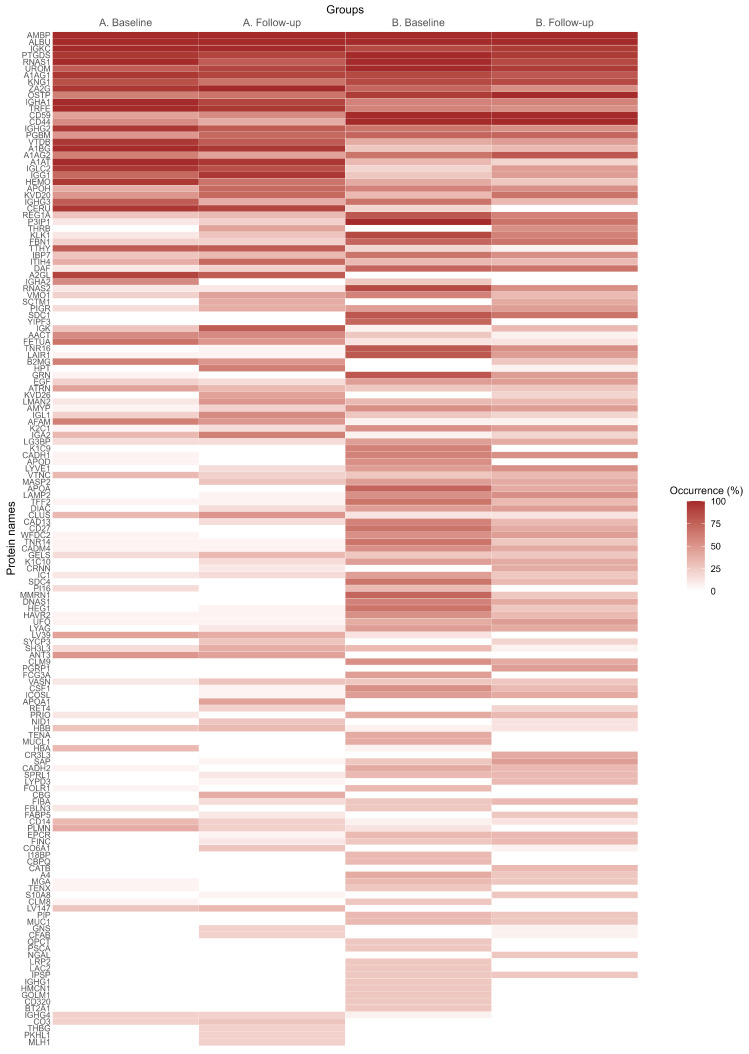
This heatmap illustrates protein distribution rates. The Y-axis lists individual proteins, and the X-axis divides groups into patient (**A**) and control groups (**B**) at baseline and follow-up. Each cell’s colour intensity represents the percentage of proteins detected within each group, with higher intensities indicating greater detection rates.

The median proteinuria declined markedly from 3.1 g/24 h at baseline to 2.1 g/24 h at follow-up, although this change was not statistically significant (*p* = 0.266). However, the median emPAI significantly decreased from 86 at baseline to 44.8 at follow-up (*p* = 0.001). Urine proteomics analysis revealed substantial differences in protein distributions between the control and CKD groups, as well as between baseline and follow-up, as illustrated in the heatmap (Figure 2). This comparison highlights statistically significant disparities in the number of proteins detected and the emPAI for total proteins between groups at baseline but not at follow-up (Table 1). At follow-up, the number of detected proteins was higher in the patient group compared to baseline (Table 1 and Figure 2).

### 3.3. Correlated Urinary Proteins with Kidney Function

The follow-up analysis of correlations between urinary proteins and eGFR showed varied associations across the cohort (Table 3). At baseline, VTDB and IGHA1 were negatively correlated with eGFR; however, only IGHA1 retained a significant correlation at follow-up. Among the proteins with positive correlations at baseline, RNAS1, PTGDS, CD44, A1AG2, VTNC, and CD59 were significant, though only RNAS1 and CD59 remained positively correlated with eGFR at follow-up.

In the subgroup analysis (Table 4), participants with CKD showed a dynamic correlation pattern with some proteins. For example, VTDB was initially negatively correlated with eGFR in CKD patients but shifted to a significant positive correlation at follow-up, indicating that its association with kidney function may change with disease progression. CERU was positively correlated with eGFR at baseline in the CKD group, though this was not observed at follow-up. In control participants, A1BG was positively correlated, and ATRN was negatively correlated with eGFR at baseline but not at follow-up.

### 3.4. Associations of Proteins with Kidney Function

**Figure 3:**
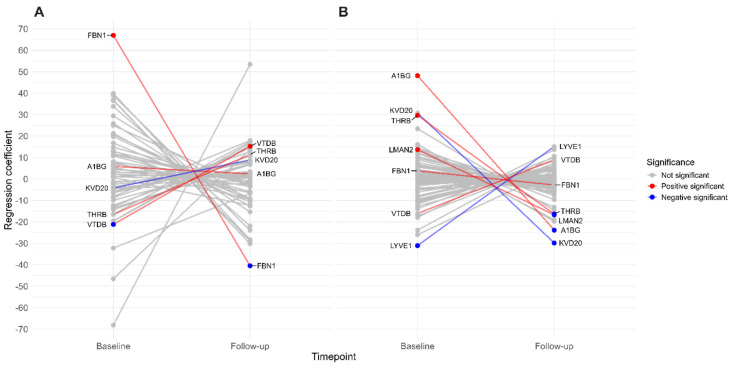
Interaction plot showing the association between the eGFR and emPAI of proteins. Regression analysis was conducted in patient (**A**) and control (**B**) groups. Only proteins with statistically significant coefficients are displayed. Please refer to Supplementary Figure S3 for the results, including all proteins. Significant interactions are colour-coded in both groups for comparison, although not all proteins had significant coefficients in both groups.

Following correlation analyses, we conducted separate regression analyses to assess the associations between proteins and eGFR within CKD and control groups, examining baseline values and their changes over time (Figure 3). In the CKD group, FBN1 showed a strong positive baseline coefficient with eGFR; however, at follow-up, it demonstrated a negative interaction coefficient with eGFR. VTDB also displayed an interesting pattern in CKD patients: its baseline coefficient was negative, while at follow-up, it exhibited a positive interaction coefficient with eGFR. In control participants, LMAN2, THRB, and A1BG had positive baseline coefficients with eGFR, while LYVE1 had a negative baseline coefficient. At follow-up, the interaction coefficients for these proteins were negative, except for LYVE1, which was positive and did not reach statistical significance over time.

**Figure 4:**
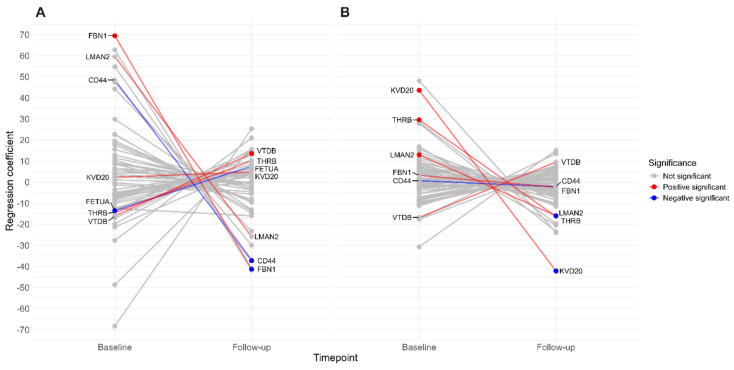
Interaction plot showing the association between eGFR and emPAI of proteins adjusted for proteinuria. Regression analysis was conducted in patient (**A**) and control (**B**) groups. Only proteins with statistically significant coefficients are displayed. Please refer to Supplementary Figure S4 for results, including all proteins. Significant interactions are colour-coded in both groups for comparison, although not all proteins had significant coefficients in both groups.

An additional regression analysis was performed to examine the relationship between the eGFR and emPAI of proteins in CKD patients and control participants, adjusting for proteinuria (Figure 4). FBN1 had a positive baseline coefficient in CKD patients, but the follow-up interaction coefficient was negative. Similarly, CD44 displayed a positive baseline association with eGFR without reaching statistical significance but showed a negative coefficient at follow-up. Also, FETUA displayed a negative baseline association with eGFR but showed a positive coefficient at follow-up without reaching statistical significance. VTDB demonstrated a negative baseline relationship with eGFR without statistical significance. At the same time, its follow-up coefficient was positive, which is consistent with previous findings of positive follow-up associations in CKD patients. In the control group, KVD20 and LMAN2 exhibited positive baseline coefficients and negative follow-up interaction coefficients with eGFR. THRB also demonstrated a positive baseline association with eGFR but had a negative follow-up interaction coefficient, though this change did not reach statistical significance over time.

### 3.5. Comparison of Biomarker Levels Between Baseline and Follow-Up

To assess changes over time, a quantitative comparison of biomarker levels between baseline and follow-up was conducted using emPAI values (Table 5). VTDB and FETUA showed marked increases among the four biomarkers, while CD44 and FBN1 demonstrated decreases.

## 4. Discussion

In this follow-up study, the 24-h urinary proteome of 18 CKD patients with stages 1–3 and 15 healthy controls was analyzed using label-free quantitative proteomics. By examining 24-h urine samples instead of spot collections [21], we captured a more complete picture of proteomic changes over time. We applied regression models adjusted for proteinuria to examine the nuanced relationships between urinary protein levels and kidney function. This approach revealed significant associations between the emPAI of specific proteins and eGFR, providing a deeper understanding of protein dynamics in CKD progression.

Proteinuria is a hallmark of kidney disease, resulting from a disrupted glomerular filtration barrier and/or the renal tubules’ impaired reabsorption of filtered proteins. The excessive filtration of proteins such as albumin and light chains can overwhelm PTECs, triggering cellular stress responses, inflammation, and fibrosis. This pathological process contributes to progressive kidney damage and plays a critical role in CKD progression [6]. Despite its clinical significance, routine diagnostics typically focus on limited urinary proteins, including albumin, potentially overlooking a broader spectrum of urinary proteins that could offer valuable prognostic insights. By assessing a comprehensive urinary proteome, as performed in our study, we can identify additional protein markers that reflect underlying pathophysiological changes and provide a more detailed understanding of kidney function dynamics in CKD patients.

A higher number of proteins was observed in controls compared to CKD patients at baseline, while the number of detected proteins increased from 171 at baseline to 285 at follow-up in the CKD group. This increase can be attributed to a reduction in proteinuria levels, from a median of 3.1 g/24 h to 2.1 g/24 h in the CKD group. Higher levels of proteinuria often lead to an increase in abundant proteins such as albumin, which can have a masking effect, making it challenging to identify low-abundance proteins [22,23].

At baseline, the VTDB (the vitamin D-binding protein) displayed a negative correlation with kidney function in the whole cohort and the CKD group separately based on Spearman analysis, although this was statistically significant only in the CKD group. Additionally, VTDB was the only protein to exhibit consistent negative associations in the regression models within the CKD group despite losing statistical significance after adjustment for proteinuria. Moreover, several more proteins showed associations in the regression models at baseline. Notably, FBN1 (Fibrillin-1) was positively associated with eGFR, while FETUA (Fetuin-A) showed a strong negative association in the CKD group. In a previous study with a larger sample size, VTDB and FETUA exhibited negative associations with eGFR, even after accounting for proteinuria [10], suggesting that these proteins may indicate early underlying kidney dysfunction at baseline.

At follow-up, among the proteins positively correlated with kidney function, VTDB exhibited consistent positive associations with eGFR, even after adjustment for proteinuria in contrast to its baseline inverse relationship. This suggests possible changes in its role as kidney function evolves. This shift from negative to positive associations of VTDB with eGFR suggests its potential as a marker of kidney function improvement in CKD patients. However, this may not indicate structural improvement. Furthermore, CD44 (CD44 protein) and FBN1 were negatively associated with eGFR at follow-up, in contrast to their baseline positive relationships, which may reflect a decline in kidney function as the disease progresses in the CKD group. These findings underscore the potential of VTDB, FBN1, and CD44 as prognostic biomarkers, offering new insights into their roles in CKD progression and their relevance for clinical risk assessment.

The comparison of biomarker levels between baseline and follow-up further supports the evolving roles of these proteins in CKD progression. VTDB showed a 138% increase in emPAI levels, with a shift to a positive association with eGFR, potentially reflecting improved tubular handling or adaptive processes in response to the reduced proteinuria observed in our follow-up study. Similarly, FETUA levels increased by 68.97%, although FETUA did not exhibit a significant association with eGFR at follow-up. In contrast, CD44 and FBN1 demonstrated decreases of −34.15% and −13.4%, respectively; these proteins are involved in renal tubular and extracellular matrix pathologies [24,25], though the implications of their reduced urinary levels in CKD progression require further investigation.

VTDB is the transporter of circulating vitamin D. With a molecular mass of 52 kDa, it is filtered through the glomerular barrier and reabsorbed by PTECs [26]. Its elevation during proteinuria is mainly due to reduced tubular reabsorption. In a previous study, Kalantari et al. identified VTDB as a predictive biomarker for the severity of IgA nephropathy; however, in their study, kidney biopsy was used for disease severity classification and predictive analysis without the follow-up validation of association dynamics [27]. Another follow-up study involving microalbuminuric patients suggested that VTDB is a urinary biomarker of tubulointerstitial damage; however, it was not associated with eGFR [28].

FBN1 is a microfibrillar protein known as a fibrotic kidney tissue scaffold component in CKD patients [25]. Its urinary excretion and relationship with kidney function have not been reported. CD44 is a transmembrane glycoprotein reported to be reduced in urine in conditions such as acute kidney transplant rejection and IgA nephropathy [29,30], which aligns with our findings where it was reduced but positively associated with eGFR in the CKD group [5,10].

Our study has several limitations. First, the sample size was small, limiting the strength of the conclusions. While our findings provide valuable preliminary data, this study serves as an exploratory and hypothesis-generating investigation. We acknowledge that the follow-up interval and sample size may limit the detection of long-term changes in CKD progression. Future studies with larger multicenter cohorts and extended follow-up periods are needed to validate and expand upon our observations. Additionally, there was a gender imbalance between the two groups, which may have affected the results; thus, future studies should aim for a more balanced gender distribution. Second, we only included patients with early-stage CKD, which may not reflect the changes in the later stages of the disease. The identified protein associations might differ in patients with later stages of CKD. Third, although we found significant associations between changes in eGFR and specific proteins using emPAI values, we acknowledge their semiquantitative nature. Future studies with absolute quantification methods (e.g., ELISA or targeted mass spectrometry) are needed to validate these findings and assess their diagnostic potential. Furthermore, during the analysis, we did not remove high-abundance proteins, which could have masked the detection of proteins present in lower amounts, especially at baseline when proteinuria was higher. Lastly, a kidney biopsy was unavailable to validate the disease diagnosis.

## 5. Conclusions

In this follow-up study, we observed notable shifts in the associations of urinary proteins with kidney function over time. VTDB transitioned from a negative association at baseline to a positive association at follow-up, while FBN1 and CD44 shifted from positive to negative. These dynamic changes in the urinary proteome suggest the evolving roles of these proteins in CKD progression, potentially indicating damage and/or recovery processes. These findings support the prognostic value of urinary proteins in monitoring disease trajectory. Further studies with larger cohorts and validation using targeted immunoassays are needed to strengthen these observations and confirm their clinical relevance.

## Figures and Tables

**Table 1:** Clinical and biochemical characteristics of participants.

Parameters	Baseline		*p*-Value	Follow-Up		*p*-Value
	CG, *n* = 15	CKD, *n* = 18		CG, *n* = 15	CKD, *n* = 18	
Demographics						
Age, year	37 ± 8	39 ± 11	0.563	38 ± 8	39 ± 11	0.612
Gender, female, *n* (%)	11 (73)	7 (39)		11 (73)	7 (39)	
eGFR, mL/min/1.73 m^2^	112.9 (105.6–122.9)	66.7 (53.7–110.4)	**0.001**	110 (106.9–118)	76 (55–100.4)	**0.002**
CKD etiology						
Glomerular, *n* (%)		14 (77.8)			14 (77.8)	
Transplant, *n* (%)		1 (5.6)			1 (5.6)	
Diabetic, *n* (%)		1 (5.6)			1 (5.6)	
Lupus, *n* (%)		1 (5.6)			1 (5.6)	
CKD of unknown etiology, *n* (%)		1 (5.6)			1 (5.6)	
Comorbidities						
Hypertension, *n* (%)	1 (7)	12 (67)		1 (7)	12 (67)	
Anemia, *n* (%)	1 (7)	5 (28)			3 (16.7)	
Laboratory data						
WBC 10 × 10^9^/L	6.1 (5.2–6.2)	7.5 (5.7–8.9)	0.06	6.2 (5.5–7)	7.6 (5.3–9.4)	0.2
PLT 10 × 10^9^/L	251 ± 42	273.7 ± 42	0.082	242.6 ± 50.4	299.9 ± 75.8	**0.027**
RBC 10 × 10^12^/L	4.8 ± 0.6	4.4 ± 0.6	0.041	4.7 ± 0.6	4.5 ± 0.5	0.61
HGB g/L	134.2 ± 18	125.8 ± 21	0.241	133.8 ± 18.2	131.9 ± 18.3	0.783
ESR, mm/h	9.6 (6–12)	21 (14–30)	**<0.001**	11.3 (6–15)	17.3 (7.5–28)	0.202
Serum total protein, g/L	69.8 (67.5–72.6)	54.6 (45.8–65.2)	**<0.001**	70.5 ± 3.1	62.3 ± 6.9	**<0.001**
Total cholesterol, mmol/L	4.6 ± 0.6	6 ± 1.7	**0.01**	4.7 ± 0.6	5.5 ± 1.8	0.23
Serum creatinine, µmol/L	63.7 (53.2–71.8)	106.4 (66.7–148.5)	**0.008**	65 (54.5–69.8)	111.1 (71–141)	**0.003**
Serum uric acid, µmol/L	295.4 (245.9–313.5)	376.9 (297–406)	**0.044**	328.9 (252.4–483.5)	408.7 (339.7–502.2)	**0.045**
Total bilirubin, µmol/L	8.9 (5.23–11.9)	8.3 (4–9.1)	0.244	8.1 (6.3–10.1)	9.4 (4.8–14.5)	0.83
Serum urea, mmol/L	4.2 (3.6–4.7)	6.5 (3.9–8.7)	**0.019**	4.5 (3.4–4.9)	7.4 (4.9–9.9)	**0.003**
Serum glucose, mmol/L	5.2 (4.9–5.63)	6.4 (4.7–5.4)	0.715	5.4 ± 0.74	5.1 ± 1.6	0.086
Urinalysis of 24-h urine samples						
Proteinuria, g/24 h	0.1 (0.07–0.15)	3.1 (1–4)	**<0.001**	0.12 (0.08–0.14)	2.1 (0.5–3.51)	**<0.001**
Proteomic data of 24-h urine samples						
Detected proteins, *n*	271	171		252	285	
emPAI for total protein	47 (29.3–56.3)	86 (60.6–105.5)	**<0.001**	34.2 (9.2–46.5)	44.8 (25.7–54.6)	0.135

Significant values are in bold. CKD: chronic kidney disease; CG: control group; emPAI: exponentially modified protein abundance index; eGFR: estimated glomerular filtration rate; ESR: erythrocyte sedimentation rate; HGB: hemoglobin; PLT: platelet; RBC: red blood cells; WBC: white blood cells.

**Table 2:** Representation of participants by the KDIGO 2012 guideline during follow-up.

Predicting the prognosis of CKD by eGFR and proteinuria categories: KDIGO 2012				Persistent proteinuria categories Description and range		
				A1	A2	A3
				Normal to mildly increased	Moderately increased	Severely increased
				<150 mg/24-h	150–499 mg/24-h	≥500 mg/24-h
eGFR categories (mL/min/1.73 m m^2^) Description and range	G1	Normal or high	≥90	B 10 (30.3%) F 10 (30.3%)	B 5 (15.1%) F 6 (18.2%)	B 6 (18.2%) F 4 (12.1%)
	G2	Mildly decreased	60–89	B 1 (3.0%) F 4 (12.1%)	B 0 F 0	B 3 (9.1%) F 3 (9.1%)
	G3a	Mildly to moderately decreased	45–59	B 0 F 0	B 1 (3.0%) F 0	B 6 (18.2%) F 4 (12.1%)
	G3b	Moderately to severely decreased	30–44	B 1 (3.0%) F 2 (6.1%)	B 0 F 0	B 0 F 0
	G4	Severely decreased	15–29			
	G5	Kidney failure	<15			

Green represents a low risk; yellow represents a moderately increased risk; orange represents a high risk; red represents a very high risk. B: baseline; F: follow-up; KDIGO: Kidney Disease: Improving Global Outcomes.

**Table 3:** Correlations of emPAI of proteins with eGFR in the whole cohort.

Urinary Proteins	Baseline			Follow-Up		
	Spearman’s Rho	*p*-Value	N of Obs.	Spearman’s Rho	*p*-Value	N of Obs.
VTDB	−0.47	**0.024**	23	0.099	0.67	21
IGHA1	−0.11	0.57	27	−0.44	**0.033**	24
VTNC	0.66	**0.04**	10	0.067	0.86	9
A1AG2	0.57	**0.008**	21	0.017	0.94	19
RNAS1	0.56	**<0.001**	33	0.6	**0.002**	26
PTGDS	0.52	**0.002**	32	0.21	0.26	29
CD44	0.49	**0.013**	25	−0.13	0.56	21
CD59	0.48	**0.021**	23	0.49	**0.015**	24

Significant values are in bold. All *p*-values reported are unadjusted.

**Table 4:** Correlations of emPAI of proteins with eGFR in two groups.

Urinary Proteins	Baseline						Follow-Up					
	CKD Group			Control Group			CKD Group			Control Group		
	Spearman’s Rho	*p*-Value	N of Obs.	Spearman’s Rho	*p*-Value	N of Obs.	Spearman’s Rho	*p*-Value	N of Obs.	Spearman’s Rho	*p*-Value	N of Obs.
VTDB	−0.48	0.051	17	−0.54	0.26	6	0.59	**0.029**	14	0.68	0.095	7
CERU	0.52	**0.032**	17	−1	0.16	3	0.24	0.36	16	NA	NA	NA
A1BG	0.43	0.077	18	1	**0.024**	4	0.39	0.12	17	0.4	0.59	4
ATRN	0.19	0.64	8	−1	**0.024**	4	−0.89	**0.026**	6	−0.4	0.59	4
CD59	0.19	0.64	8	0.18	0.52	15	0.64	**0.05**	10	0.25	0.39	14

Significant values are in bold. All *p*-values reported are unadjusted.

**Table 5:** Percentage changes in biomarker levels between baseline and follow-up.

Candidate Biomarker	Baseline (Mean emPAI)	Follow-Up (Mean emPAI)	Percentage Change
VTDB	0.34	0.81	138
FETUA	−0.58	−0.98	68.97
CD44	−3.63	−2.39	−34.15
FBN1	−5.22	−4.52	−13.4

## Data Availability

The datasets produced and/or utilized in this study, including the proteomic data, are provided as Supplementary Data S1. Additional information can be obtained from the corresponding author (A.G.) upon reasonable request.

## Supplementary Materials

The following supporting information can be downloaded at https://www.mdpi.com/article/10.3390/biom15010045/s1. Figure S1: Line plot of GFR changes over time in patient (A) and control (B) groups; Figure S2: Line plot of 24 h proteinuria level changes over time in patient (A) and control (B) groups; Figure S3: Interaction plot showing the association between the eGFR and emPAI of proteins; Figure S4: Interaction plot showing the association between the eGFR and emPAI of proteins adjusted for proteinuria.
