# Supplementary material for: Urinary Proteomic Shifts over Time and Their Associations with eGFR Decline in Chronic Kidney Disease
Source: Biomolecules. 2025 Jan 1;15(1):45. doi: 10.3390/biom15010045 (PMC11762955; doi:10.3390/biom15010045)
Supplement: Supplementary file 1 [file biomolecules-15-00045-s001.zip › Manuscript_Supplementary_Biomolecules_Final.pdf]

# Urinary proteomic shifts over time and their associations with eGFR decline in chronic kidney disease

**Supplementary materials**

**Table of contents**

**Supplementary Figure S1.** Line plot of GFR changes over time in patient (A) and control (B) groups.

**Supplementary Figure S2.** Line plot of 24-h proteinuria level changes over time in patient (A) and control (B) groups.

**Supplemenatal Figure S3.** Interaction plot showing association between eGFR and emPAI of proteins.

**Supplemenatal Figure S4.** Interaction plot showing association between eGFR and emPAI of proteins adjusted for proteinuria.

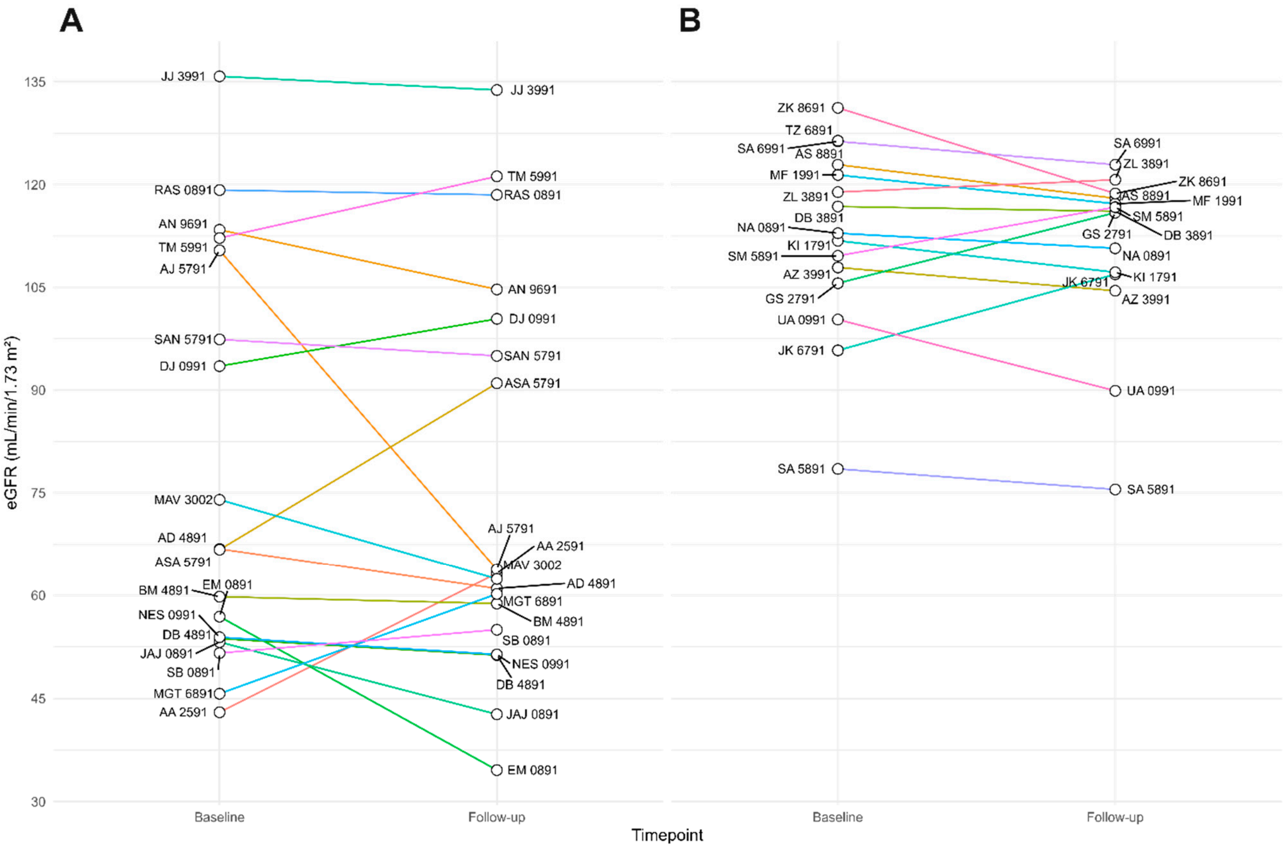


**Supplementary Figure S1.** Line plot of GFR changes over time in patient (A) and control (B) groups. The X-axis displays individual participant IDs to show GFR changes from baseline to follow-up, while the Y-axis conveys GFR values.

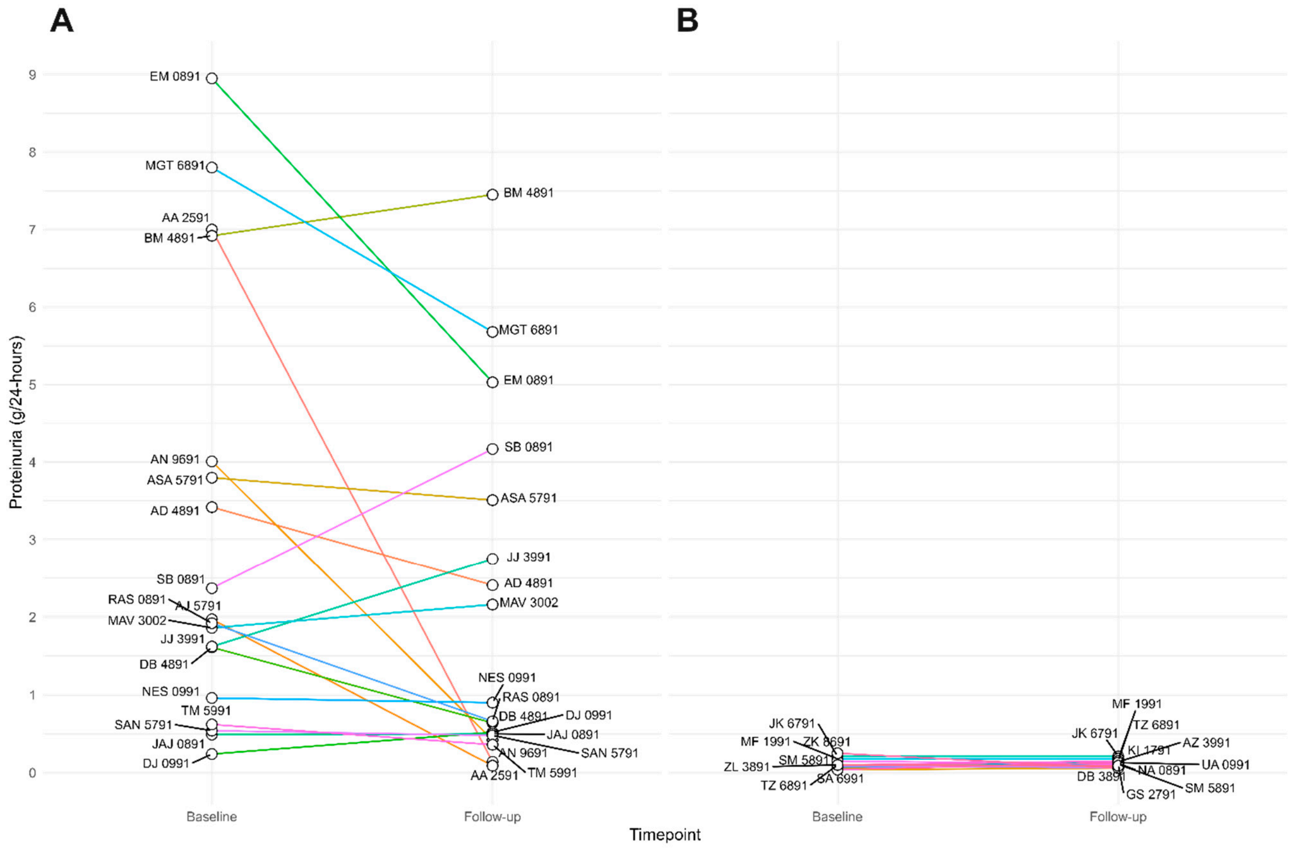


**Supplementary Figure S2.** Line plot of 24-h proteinuria level changes over time in patient (A) and control (B) groups. The X-axis displays individual participant IDs to show proteinuria changes from baseline to follow-up, while the Y-axis conveys 24-h proteinuria values.

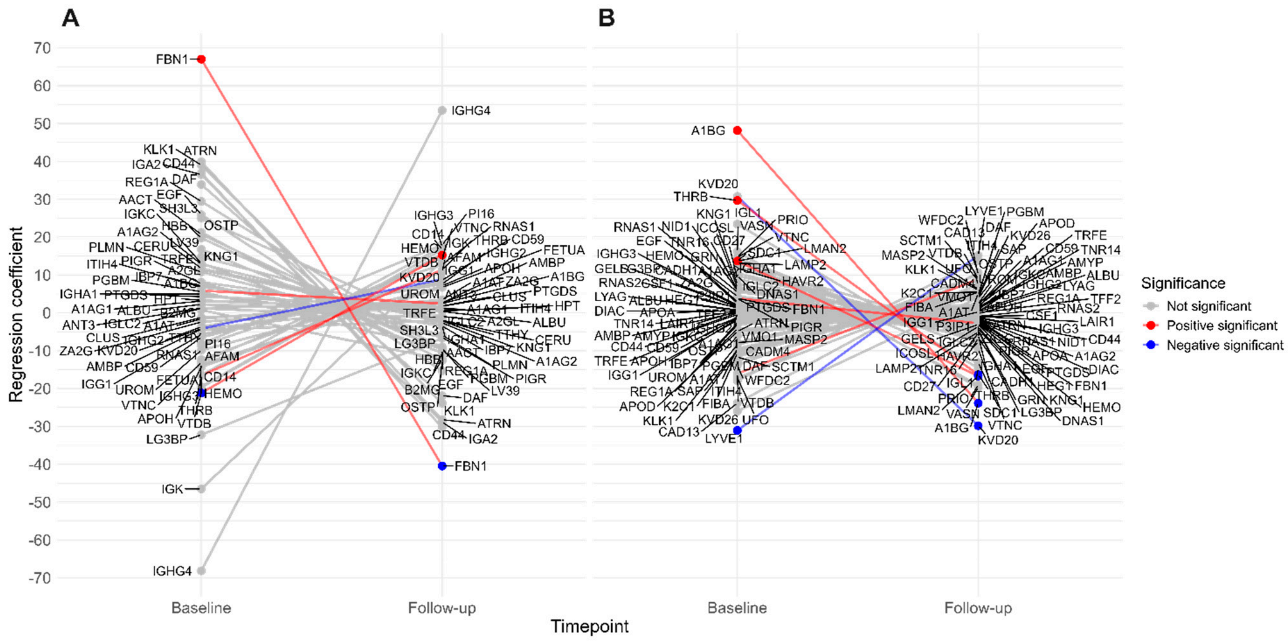


**Supplemenatal Figure S3.** Interaction plot showing association between eGFR and emPAI of proteins. Regression analysis in patient (**A**) and control groups (**B**).

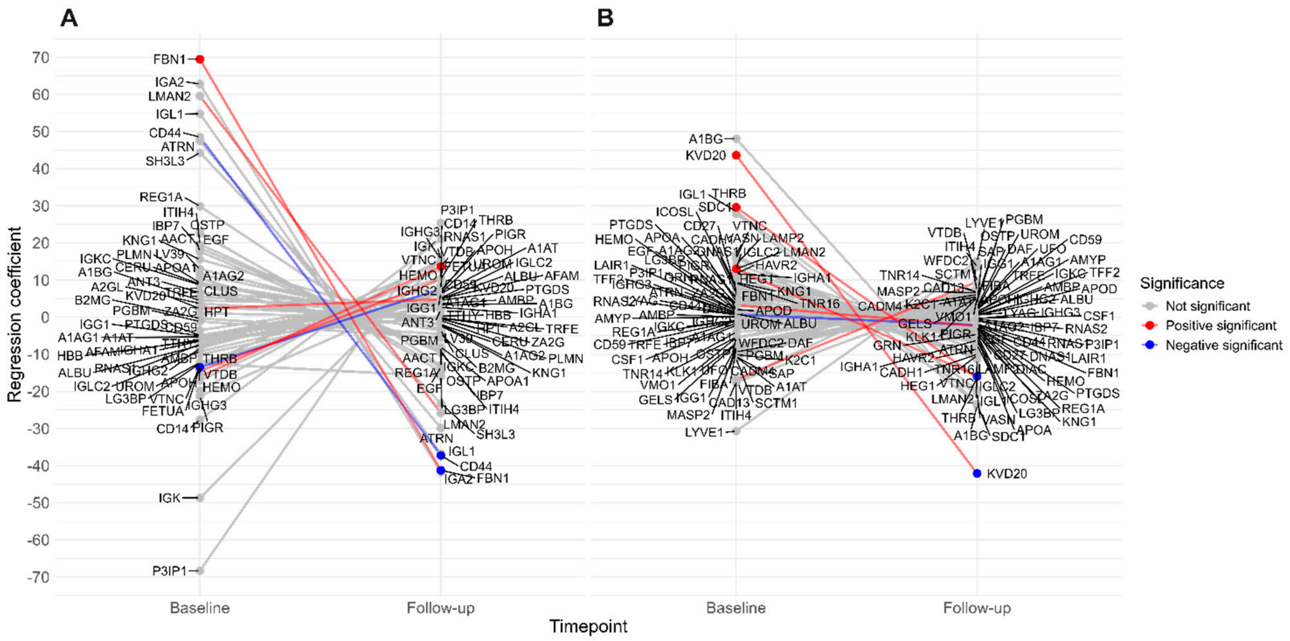


**Supplemenatal Figure S4.** Interaction plot showing association between eGFR and emPAI of proteins adjusted for proteinuria. Regression analysis in patient (**A**) and control groups (**B**).
